# Massive Exudative Pleural Effusion With Hypothyroidism: A Case Report

**DOI:** 10.7759/cureus.80683

**Published:** 2025-03-16

**Authors:** Yukiko Uehara, Noriko Ogawa, Takuma Yamoto, Nobuhide Watanabe, Keizo Kanasaki

**Affiliations:** 1 Endocrinology and Metabolism, Shimane University Faculty of Medicine, Izumo, JPN; 2 Cardiology, Shimane University Faculty of Medicine, Izumo, JPN

**Keywords:** ascites, hypothyroidism, pericardial effusion, pleural drainage, pleural effusion

## Abstract

Here, we report the case of a 79-year-old woman with massive exudative pleural effusion and hypothyroidism. The patient underwent total thyroid and parathyroidectomy during pharyngoesophagectomy for hypopharyngeal cancer. She was administered a thyroid hormone preparation (87.5 μg of levothyroxine sodium), calcium supplementation (0.5 g of calcium lactate hydrate), and active vitamin D (2 g of alfacalcidol). Four months after missing her regular medical appointment and discontinuing her medications, she developed a severe exudative pleural effusion, circumferential pericardial effusion, and mild ascites secondary to hypothyroidism. The pleural effusion, which may have been exacerbated by prolonged hypothyroidism and associated heart failure, improved with drainage and did not recur after the initiation of thyroid hormone replacement therapy. The pericardial effusion and ascites improved with hormone replacement alone. Although fluid retention associated with hypothyroidism usually improves with thyroid hormone therapy, drainage may be required to treat severe exudative effusions in diverse body cavities when diuretic treatments are insufficient.

## Introduction

Hypothyroidism can cause pericardial fluid, pleural effusion, and ascites due to heart failure, as well as exudative effusions in the body cavities due to myxedema, a severe form of hypothyroidism [1]. It also leads to symptoms associated with hypometabolism, such as fatigue, cold intolerance, weight gain, slow movements and thoughts, depression, and constipation. [2]. The prevalence of pleural effusion in patients with hypothyroidism is up to 25%, while that of pericardial effusion is 3%-37%, although severe pleural effusions are rare [3-6]. Here, we present a case of hypothyroidism with a marked right-sided predominantly exudative pleural effusion.

## Case presentation

Clinical presentation

A 79-year-old woman presented at our hospital with fatigue, shortness of breath, cold intolerance, and limb edema. She had undergone a total thyroid and parathyroidectomy 29 years previously when her pharyngoesophagus was removed to treat hypopharyngeal cancer. She was taking thyroid hormone preparations (87.5 μg of levothyroxine sodium), calcium supplements (0.5 g of calcium lactate hydrate), and an active vitamin D prodrug (2 μg of alfacalcidol). She had accidentally forgotten to visit her family doctor and stopped taking her medication four months prior to presenting at our hospital. She seemed to have forgotten the reason for and importance of taking her medication since she had initiated it approximately 30 years prior. She was admitted to our hospital with hypothyroidism, hypocalcemia, decreased oxygen saturation, marked right pleural fluid accumulation, and pitting edema of the legs. She also had a 20-year history of hypertension.

Physical examination

The patient's height was 153.7 cm, weight was 49.1 kg, and body mass index was 20.97 kg/m^2^. She had a waist circumference of 73 cm, temperature of 36.2°C, blood pressure of 156/60 mmHg, pulse of 60/min, and oxygen saturation (SpO_2_) of 93% on room air. She was conscious and had a permanent tracheal stoma. No abnormal heart sounds were recorded. Her breath sounds were decreased on the right side. Her abdomen was flat and soft, and the liver and spleen could not be palpated. Pitting edema was observed on the dorsum of the hands and lower legs.

Laboratory findings

An endocrinological examination revealed low thyroid hormone and elevated thyroid-stimulating hormone (TSH) levels: free T_3_, 0.9 pg/mL (reference range, 2.1 to 3.8 pg/mL); free T_4_, 0.4 ng/dL (reference range, 0.8 to 1.5 ng/dL); and TSH, 117.506 mU/L (reference range, 0.61 to 4.23 mU/L). Antibodies suggestive of Hashimoto's disease were negative. The intact parathyroid hormone (PTH) level was below the limit of detection (<1.0 pg/mL, reference range, 10 to 65 pg/mL), and the adjusted Ca level was low at 5.9 mg/dL (reference range, 8.8 to 10.1 mg/dL). These findings led to diagnoses of hypothyroidism and hypoparathyroidism.

Imaging studies

**Figure 1:**
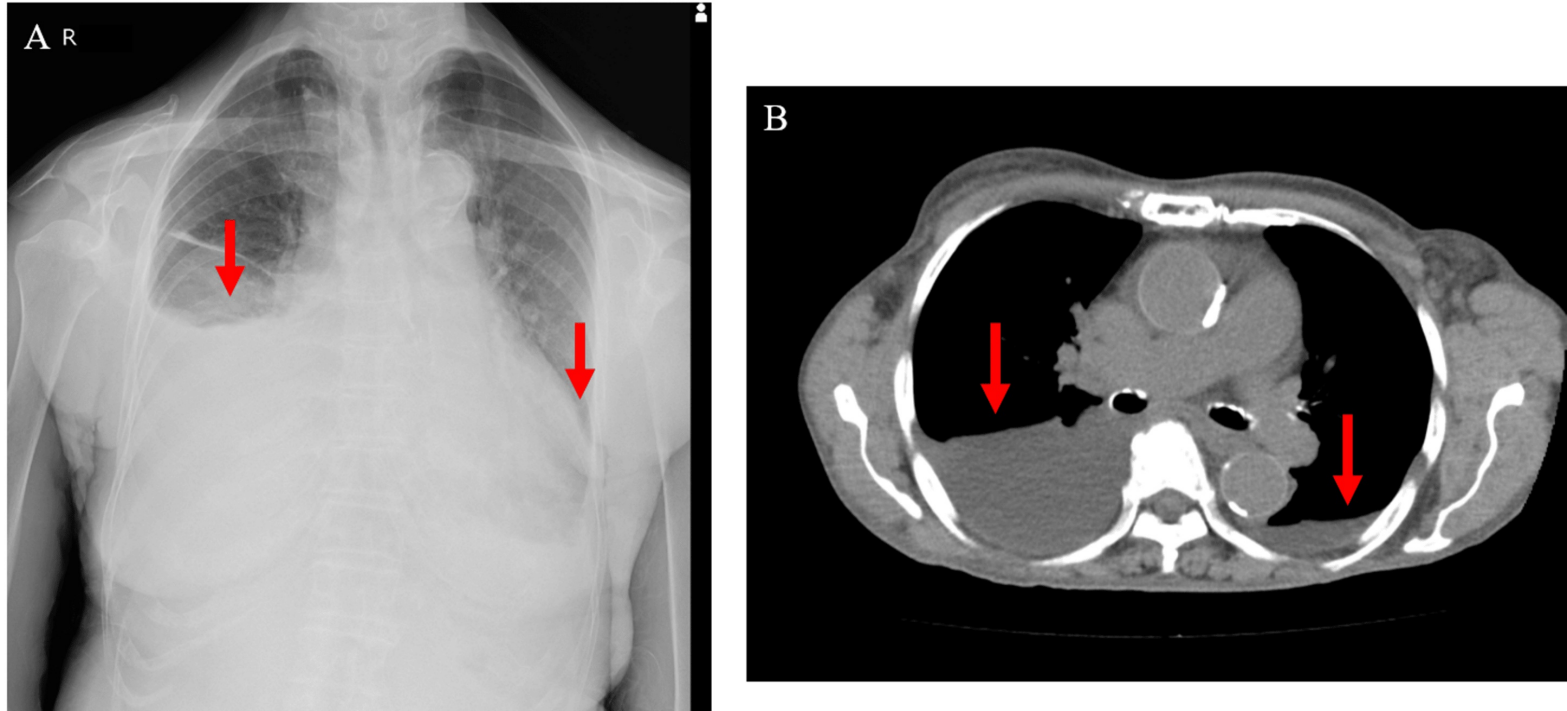
Pleural effusion (red arrow) on chest X-ray (A) and chest CT scan (B) CT: computed tomography

**Figure 2:**
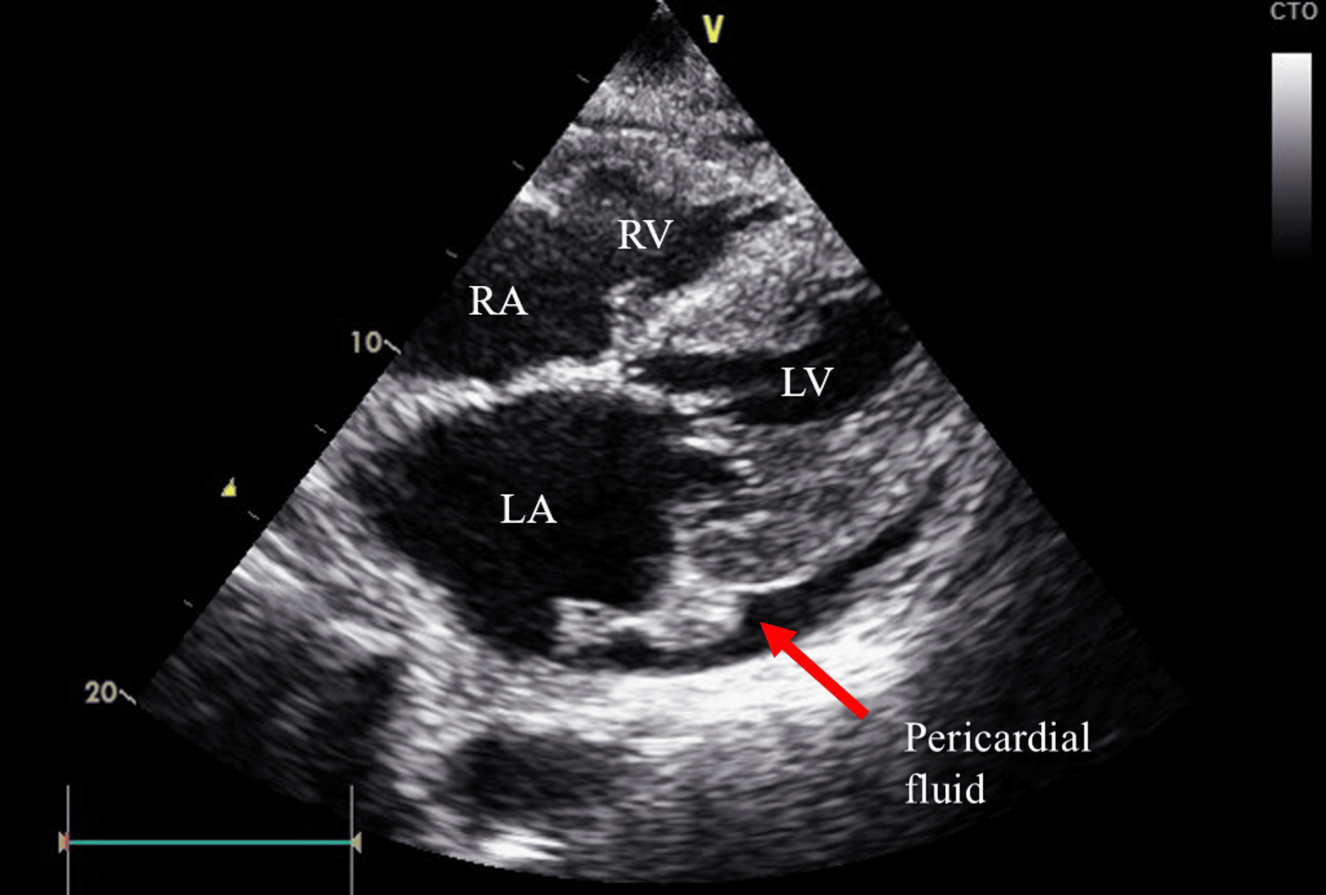
Circumferential pericardial effusion on echocardiography LA: left atrium; LV: left ventricle; RA: right atrium; RV: right ventricle

Chest radiography and computed tomography (CT) revealed predominantly right-sided pleural fluid and cardiac dilatation with a cardiothoracic ratio of 75% (Figure 1). Echocardiography revealed a circumferential pericardial effusion (Figure 2), mild mitral regurgitation, trivial tricuspid regurgitation, a 50% left ventricular ejection fraction, and impaired left ventricular dilatation. She was diagnosed with heart failure with preserved ejection fraction. A small amount of ascites was observed on abdominal CT.

Management

**Figure 3:**
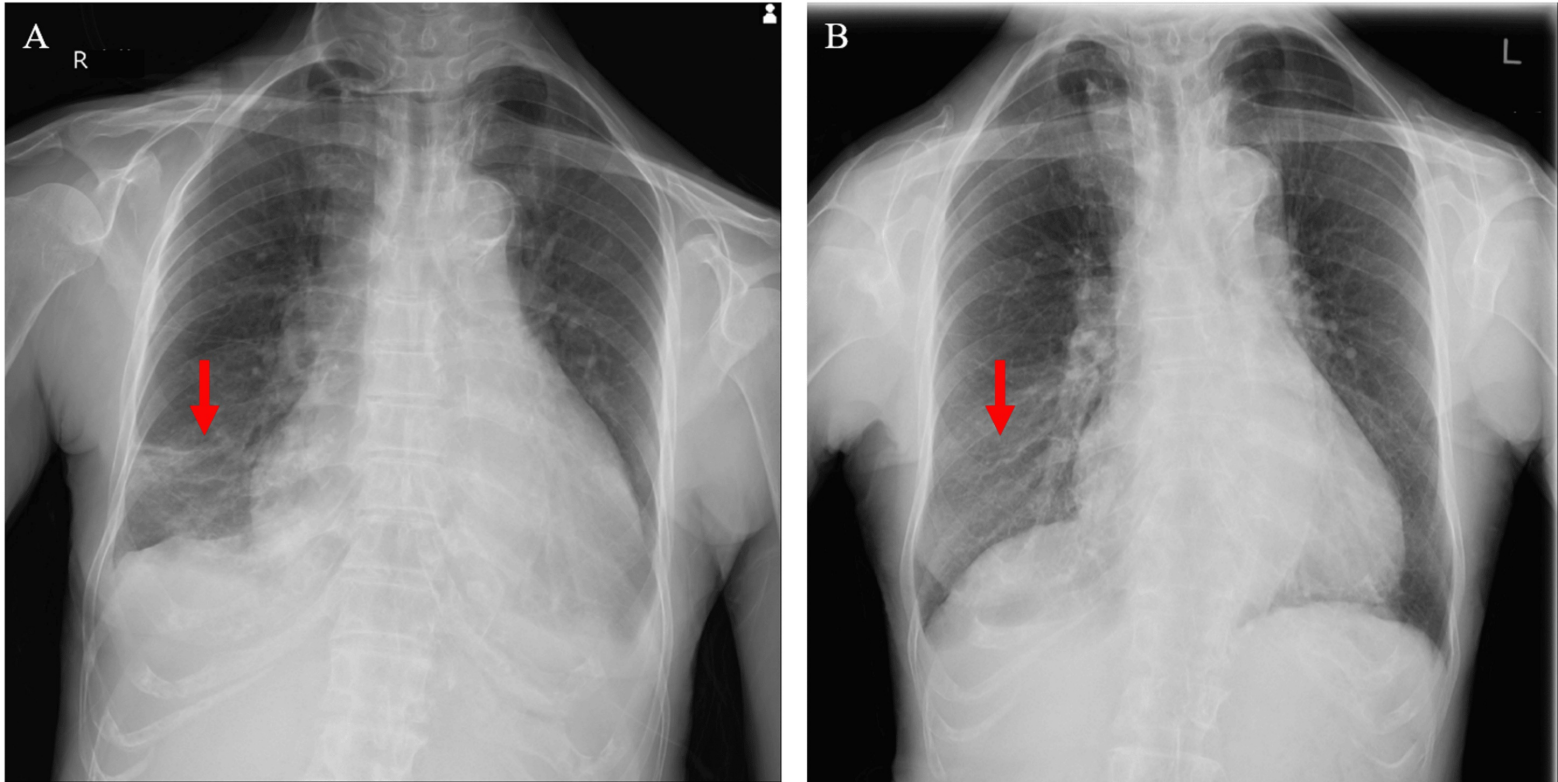
Chest X-ray after pleural drainage A: X-ray image immediately after pleural drainage. B: X-ray image taken four months later.

**Figure 4:**
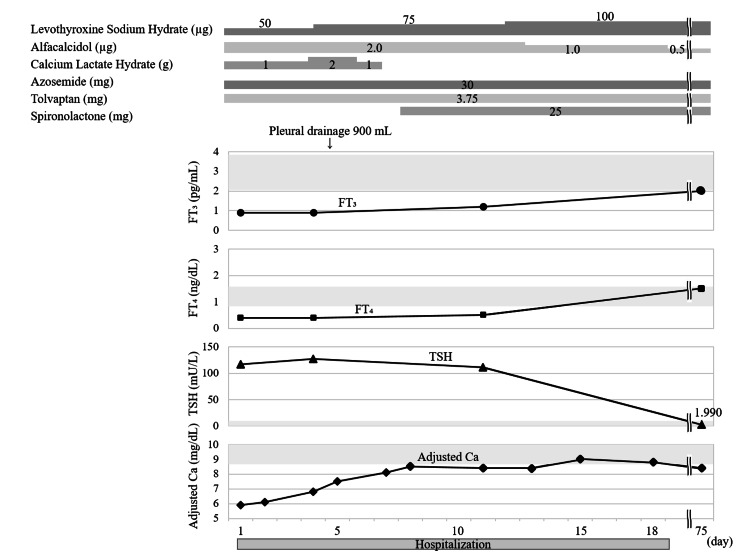
Patient's clinical course The gray area of the graph represents the reference range. Ca: calcium; FT_3_: free triiodothyronine; FT_4_: free thyroxine; TSH: thyroid stimulating hormone

The patient was treated for hypothyroidism and hypoparathyroidism with 50 μg of levothyroxine sodium, 1.0 g of calcium lactate hydrate, and 2 μg of alfacalcidol. She was administered loop diuretics (30 mg of azosemide) and a vasopressin V2 receptor antagonist (3.75 mg of tolvaptan) to treat heart failure. As the pleural effusion did not improve and the low SpO_2_ persisted despite thyroid hormone replacement, a thoracentesis (900 mL) was performed on day five of admission. The pleural effusion was exudative, with a pleural fluid/serum protein ratio >0.5 (0.625 (pleural 4.0/serum 6.4 g/dL)) and pleural fluid lactate dehydrogenase level (173 U/L) >2/3 (148 U/L) of the upper reference limit of normal (222 U/L). There were no malignant findings on CT and no malignant cells in the pleural effusion. There were no symptoms or laboratory findings suggestive of tuberculosis, rheumatoid arthritis, or systemic lupus erythematosus. There was no evidence of hepatic or renal dysfunction or anemia sufficient to cause the edema. Although the contribution of long-term hypertension to heart failure could not be completely excluded, the cardiac diastolic dysfunction and pericardial effusion improved upon thyroid hormone replacement. Chest radiography revealed no recurrence of the pleural effusion after drainage (Figure 3). We considered that the pleural effusion was related to hypothyroidism because it did not recur after the thoracentesis with the continued thyroid hormone administration. Her symptoms resolved after the thoracentesis and thyroid hormone replacement therapy. The mild ascites also improved with thyroid hormone replacement therapy. The patient’s clinical course is shown in Figure 4.

## Discussion

Here we described the case of a patient who developed a severe exudative pleural effusion with hypothyroidism after total thyroidectomy and a four-month interruption of 87.5 μg of levothyroxine sodium treatment.

Causes of pleural effusion in patients with hypothyroidism include heart failure and myxedema, and the effusion's characteristics depend on the degree of their involvement. Therefore, a pleural effusion with hypothyroidism may be transudative, exudative, or borderline between them [7]. The transudative pleural effusion may be caused by the hypothyroidism-associated heart failure. Hypothyroidism impairs myocardial relaxation owing to decreased reuptake of Ca ions via the sarcoplasmic reticulum Ca-ATPase-phospholamban system [8]. Hypothyroidism impairs vascular smooth muscle relaxation, reduces endothelial nitric oxide production, increases systemic vascular resistance, and impairs left ventricular diastolic function [9-12]. On the other hand, myxedema is a suggested cause of exudative pleural effusions. Hypothyroidism causes increased vascular permeability via vascular endothelial growth factor, albumin extravasation, and inadequate lymphatic drainage, leading to effusion leakage into the body cavities [1,13-15]. In the present case, the pleural effusion was exudative and considered myxedematous [16]. Some cases of exudative pleural effusion have been reported [17,18].

Our patient exhibited a severe pleural effusion, predominantly on the right side. In a study of heart failure, the prevalence of pleural fluid was reportedly 58% bilateral, 27% right-sided, and 14% left-sided [19]. Several theories have been proposed to explain this phenomenon; however, an experimentally proven mechanism is lacking [20]. The lymphatic vessels are reportedly thinner on the right side, while the lymphatic return is enhanced by cardiac pulsation on the left side [21]. It has also been suggested to be anatomical, such as increased hydrostatic pressure predominantly on the right side due to compression of the azygos vein and atrial dilatation causing compression of the right pulmonary vein [20,22].

The patient underwent the drainage of a 900 mL pleural effusion. Hypothyroidism with a severe pleural effusion is rare [5,6]. While the presence or volume of a pleural effusion is not reportedly related to hypothyroidism severity, left ventricular dysfunction is associated with low serum T_3_ levels [7,12]. The extravascular accumulation of albumin is reportedly evident after only two to three months of hypothyroidism [15]. The severe pleural effusion in the present case could be attributed to the prolonged period of hypothyroidism with a four-month treatment interruption. Moreover, heart failure due to impaired left ventricular dilatation associated with hypothyroidism may have contributed to the worsening of the pleural effusion.

In patients with hypothyroidism, effusions commonly occur in various body cavities rather than in a single location [1]. The present case involved a circumferential pericardial effusion and a small amount of ascites in addition to the pleural effusion. The cause of the exudative pericardial effusion is considered similar to that of an exudative pleural effusion in myxedema [4,23]. Myxedematous ascites are rare, with an incidence of <1% [24-26]. Similar to exudative pleural and pericardial effusions, the pathogenesis of myxedematous ascites involves myxedema [15,24,27]. In the present case, the pericardial effusions and ascites may have been of exudative origin, although no pericardiocentesis or paracentesis was performed.

The treatment of pleural and pericardial effusions in hypothyroidism involves normalizing the thyroid function using hormone replacement therapy [7,28,29]. Hypothyroidism-associated cardiac dysfunction can also be improved with thyroid hormone replacement therapy [30,31]. In this case, despite the gradual increase in thyroid hormone replacement therapy from a low starting dose and the administration of diuretics, the pleural effusion remained severely burdensome and the patient continued to exhibit respiratory distress and a low SpO_2_ of 90%-95%. On hospitalization day five, a 900-mL pleural effusion was drained via thoracentesis. Thyroid hormone replacement therapy was continued, and the pleural effusion did not recur. Moreover, the pericardial effusion and ascites resolved with the thyroid hormone replacement treatment.

In this case, a four-month interruption in thyroid hormone replacement therapy resulted in a severe exudative pleural effusion with hypothyroidism, circumferential pericardial effusion, and a small amount of ascites. The pleural effusion may have been exacerbated by prolonged hypothyroidism and concomitant heart failure due to impaired left ventricular dilatation. In the present case, two months after the initiation of treatment, step-up adjustments to the thyroid hormone replacement resulted in a marked improvement in thyroid function. The myxedema may have reduced intestinal absorption when the supplementation was first resumed. In cases of hypothyroidism-associated pleural effusion, diuretics may be effective if the effusion is transudative and mainly caused by heart failure. However, if the effusion accumulates in multiple other body cavities, it is likely to be exudative due to myxedema [1]. Although exudative effusion retention is generally expected to improve with thyroid hormone replacement alone, it may be difficult to improve significant exudative effusion retention in the short term using only titrated thyroid hormone replacement; therefore, drainage should be considered.

## Conclusions

Here, we presented a case of a severe exudative pleural effusion, circumferential pericardial effusion, and mild ascites associated with hypothyroidism due to a four-month interruption of thyroid hormone replacement therapy following total thyroidectomy. Hypothyroidism-induced effusions in body cavities generally improve with thyroid hormone replacement therapy. However, effusions in multiple cavities are likely to be exudative and may require drainage if thyroid hormone replacement and diuretics are insufficient.
